# Household biomass fuel use, asthma symptoms severity, and asthma underdiagnosis in rural schoolchildren in Nigeria: a cross-sectional observational study

**DOI:** 10.1186/s12890-016-0352-8

**Published:** 2017-01-05

**Authors:** Oluwafemi Oluwole, Ganiyu O. Arinola, Dezheng Huo, Christopher O. Olopade

**Affiliations:** 1 Department of Community Health and Epidemiology and the Canadian Centre for Health and Safety in Agriculture, College of Medicine, University of Saskatchewan, Saskatoon, SK Canada; 2 College of Medicine, University of Ibadan, Ibadan, Oyo State Nigeria; 3 Department of Public Health Science, University of Chicago, Chicago, IL USA; 4 Department of Medicine and The Center for Global Health, University of Chicago, 5841 S Maryland Avenue, MC 6076, Chicago, IL 60637 USA

**Keywords:** Biomass fuel, Asthma severity, Possible asthma, Underdiagnosis, Rural children, Nigeria

## Abstract

**Background:**

In 2014, the International Study of Asthma and Allergies in Childhood (ISAAC) reported that the highest prevalence of symptoms of severe asthma was found in the low- and middle-income countries (LMICs), including Nigeria. While exposure to biomass fuel use may be an important risk factor in the development of asthma, its association with asthma symptoms severity has not been well-established. The aim of this study is to extend the spectrum of environmental risk factors that may be contributing towards increasing asthma morbidity, especially asthma symptoms severity in rural schoolchildren in Nigeria and to examine possible asthma underdiagnosis among this population.

**Methods:**

Authors conducted a cross-sectional survey in three rural communities in Nigeria. Asthma symptoms were defined according to the ISAAC criteria. Information on the types of household fuel used for cooking was used to determine household cooking fuel status. Asthma symptoms severity was defined based on frequencies of wheeze, day- and night-time symptoms, and speech limitations. Logistic regression analyses were used to explore associations.

**Results:**

A total of 1,690 Nigerian schoolchildren participated in the study. Overall, 37 (2.2%) had diagnosed asthma and 413 (24.4%) had possible asthma (asthma-related symptoms but not diagnosed asthma). Children from biomass fuel households had higher proportion of possible asthma (27.7 vs. 22.2%; *p* < 0.05) and symptoms of severe asthma (18.2 vs. 7.6%; *p* = 0.048). In adjusted analyses, biomass fuel use was associated with increased odds of severe symptoms of asthma [odds ratios (OR) = 2.37; 95% CI: 1.16–4.84], but not with possible asthma (OR = 1.22; 95% CI: 0.95–1.56).

**Conclusion:**

In rural Nigerian children with asthma symptoms, the use of biomass fuel for cooking is associated with an increased risk of severe asthma symptoms. There is additional evidence that rural children might be underdiagnosed for asthma.

## Background

Roughly 334 million people worldwide suffer from asthma, and the prevalence is increasing particularly among children [1]. Reasons for this trend are currently unclear, but factors including lifestyle changes [1–3], environmental challenges (such as traffic-related pollution) [4], and tobacco smoke exposure [2, 5], dust mites [6], indoor dampness and molds [7], and pets [4] have been implicated. Additionally, household air pollution (HAP) from the use of biomass for cooking is increasingly being recognized as a risk factor for asthma and other respiratory symptoms, especially in low- and middle-income countries (LMIC) [8, 9].

While asthma remains the most common chronic disease in children and accounts for a substantial burden of healthcare costs globally [1], the severity of symptoms in affected children is variable; some children are affected to a limited degree, while others suffer from severe symptoms with frequent exacerbations and possible hospitalization [10]. Hence, the assessment of the degree of symptoms severity and associated risk factors has become an integral part of childhood asthma management guidelines [10]. Several studies have shown that using open fire for cooking was adversely associated with an increased risk of asthma and asthma-related symptoms [8, 9, 11, 12]. However, few studies have investigated the role of exposure to smoke from biomass use on the degree of asthma and asthma symptoms severity or the frequency of symptom exacerbations.

The International Study of Asthma and Allergies in Childhood (ISAAC) estimated that the prevalence of asthma symptoms among children in Nigeria has risen from 10.7% in 1999 to approximately 20% in 2014 [1]. Despite the increasing prevalence of symptoms suggestive of asthma, the prevalence of physician-diagnosed asthma among children also remains relatively low [13]; this indicates potential underdiagnosis of asthma. Though some studies have investigated the relationships between asthma, lung health, and biomass smoke exposure in Nigeria [14–18], none have investigated the potential role of biomass fuel use on asthma symptoms severity.

With over 70% of rural Nigerian households relying on biomass fuels for cooking [19], an urgent need exists for broadening the understanding of the link between household biomass fuel use and symptoms severity in children with asthma or asthma-related symptoms. Thus, a cross-sectional survey was performed to evaluate possible relationships between household biomass fuel use and the degree of asthma symptoms severity in rural Nigerian schoolchildren. Furthermore, the authors sought to determine potential asthma underdiagnosis in this population by assessing the proportion of children with possible asthma (defined as children reporting combinations of symptoms suggestive of asthma, but who have not been physician-diagnosed with asthma).

## Methods

### Study population

The study was conducted among schoolchildren (aged 6–21 years) from 3 similar, contiguous rural communities in southwest Nigeria: Aba-Nla, Eruwa, and Igbo-Ora, all of which are located 40 to 100 km from Ibadan, a major urban center. The major source of occupation for households in these communities was farming. Hence, the majority of households are largely dependent on biomass fuel for daily cooking and energy needs; and spend, on average, five hours per day cooking with biomass fuels [16]. As a result, women and children receive substantial daily biomass smoke exposure. All 16 schools in these communities (9 secondary schools, 7 primary schools) were approached for the study. A total of 2,315 study packages, including an information letter and survey were distributed to parents through the schools for completion. Surveys were then returned to the school where they were collected by research staff. Data from 1,690 questionnaires (those which were completed and returned to schools for pick-up) form the basis of this report.

### The survey

The ISAAC questionnaire, with some modifications based on the objective of the study, was administered [20] for completion by the parent of each participating student. In addition to completing the validated asthma and respiratory symptoms portions of the modified ISAAC questionnaires, parents or guardians also completed questions on household fuel type use for cooking. The questionnaire was translated from English to Yoruba (the common language in the community) and was then reverse-translated for accuracy at the Department of Linguistics at the University of Ibadan. The translated version of this questionnaire was pilot-tested in our initial study in similar rural communities [16] and adjusted after discussions with other health workers and community representatives.

### Operational definition of variables

Information on respiratory health outcomes of physician-diagnosed asthma and current wheeze was ascertained from the modified ISAAC questionnaire. The inclusion criteria for diagnosed asthma (probable asthma), possible asthma, and no asthma are presented in Table 1. “Physician-diagnosed asthma” was operationally defined as a positive response on the questionnaire to: 1) child had a history of physician-diagnosed asthma, 2) child had any episode of asthma in the past 12 months, and 3) child had taken prescribed asthma medication in the past 12 months. Children with “possible asthma” were defined as children with positive responses to wheeze or whistling symptoms in the chest, dry cough, and activity limitation, but who had never been diagnosed with asthma by a physician or other health professional. Current wheeze status was assigned with the question: “Has your child ever had wheeze or whistling in the chest in the past 12 months?” Additionally, symptoms of severe asthma were defined as children with current wheeze who in the past 12 months had 1) 4 or more attacks of wheeze, 2) had 1 or more night sleep disturbance per week, or 3) had experienced wheeze that was severe enough to prevent the child from completing 1–2 words at a time between breaths [9]. Based on the combination of 2 or more of these characteristics among the wheezing children, symptom severity was classified into “mild/moderate persistent asthma symptoms” and “severe persistent asthma symptoms”.

**Table 1:** Asthma and asthma-like symptoms questions from survey used to classify children into asthma groups

1.	Has a doctor ever said your child has asthma?
1a.	If “yes” has this been in the past 12 months?
2.	*In the last 12 months*, has your child taken asthma medication prescribed by a doctor?
3.	Has your child ever had wheeze or whistling sound in the chest?
3a	If “yes” has this happened in the past 12 months?
4	*In the last 12 months*, has your child's sleep been disturbed due to breathing problems (e.g., wheezing or whistling in the chest, coughing, shortness of breath, chest tightness)
5.	*In the last 12 months*, has your child's chest sounded wheezy or coughed during or after exercise when he or she did not have a cold?

Doctor-diagnosed asthma (probable asthma): Primarily “*Yes*” to questions 1–2 and “*Yes*” to at least one question between questions 3 and 5; Possible asthma (“At-Risk”): “*Yes*” to one or a combination of two or more questions between questions 3 and 5; No asthma: Response of “*No*” to all questions

Information on biomass smoke exposure was obtained for each child based on household cooking fuel types. Each parent/guardian responded to the question: “What fuel type is mainly used for cooking in your house?” Parent/guardians then indicated which fuel(s) were used in the household from the following list: “cow dung/animal residue,” “firewood,” “charcoal,” “liquefied petroleum gas (LPG),” “electricity,” and “other.” These responses were used to group children into 2 distinct categories; children from parents choosing cow dung/crop residue, firewood, charcoal, or a combination of 2 or more of these household fuel types were classified as “children from biomass fuel households,” and the rest were classified as children from “no biomass fuel households”.

Finally, information on covariates of importance such as age, sex, body mass index (BMI), maternal levels of education, passive tobacco smoke exposure, and pet ownership were obtained. BMI was calculated as weight in kilograms divided by height in centimeters as reported on the questionnaire. Childhood overweight status was defined according the international cut-off value (equivalent to a body mass index of, or greater than, 25 kg/m^2^ [21]. The following variables were included in the multivariable regression models: mother’s education level, pet ownership, exposure to environmental tobacco smoke, sex, BMI, and age [22–25].

### Statistical analysis

Data processing and statistical analyses were performed using Statistical Package for the Social Science (SPSS) version 23 (SPSS Inc. Armonk, NY: IBM Corp.) and the Statistical Analysis System (SAS) version 9.4 (SAS Institute Inc., Cary, NC, USA). An alpha level of 0.05 defined statistical significance. Categorical demographics, environmental, and respiratory symptoms, (including severity categories) were compared between children from the two biomass user groups using the *χ* ^*2*^ test, while continuous variable was compared using independent *t*-tests. Binary and multivariate logistic regression analyses were performed to assess the relationships between household cooking fuel types and possible asthma as well as asthma symptoms severity among children with wheezing symptom. Variables were included in the multivariate models based on statistical significance in the univariate analysis (*p* ≤ 0.25) and biological/clinical importance. The strengths of the associations were assessed using odds ratios (ORs) with 95% confidence intervals (95%CI).

## Results

Of the 1,690 children studied, 865 (51.1%) were from households using biomass fuels for cooking and 825 (48.9%) were from households using cleaner fuels. Key demographic and environmental characteristics by household’s fuel status are presented in Table 2. Sex distributions were similar between the 2 groups and children from biomass fuel households were about 0.6 years younger on average than children from households using cleaner fuels. There were significant differences between the 2 groups for mother’s levels of education and a higher proportion of children from no biomass fuel households were more likely to be overweight. When comparing environmental characteristics, only exposure to pets (cat and dog) differed significantly between the 2 groups (*p* = 0.031).

**Table 2:** Personal and environmental characteristics of children by household biomass fuel status

	Overall (*n* = 1690)	Biomass fuel households (*n* = 865)	No biomass fuel households (*n* = 825)	*P* value
Demographics				
Mean age, years (±SD)	13.6 (2.7)	13.3 (2.7)	13.9 (2.6)	<0.001
Sex, *n* (%)				
Male	873 (51.7)	433 (50.1)	440 (53.3)	0.178
Mother’s level of education, *n* (%)				
≥ High school	200 (14.6)	77 (11.0)	123 (18.4)	<0.001
Body mass index (BMI), *n* (%)				
Overweight	26 (1.5)	6 (0.7)	20 (2.4)	0.004
Environmental exposure, *n* (%)				
Parental smoke	13 (0.8)	8 (0.9)	5 (0.6)	0.453
Environmental tobacco smoke	11.2	12.6	9.7	0.058
Pet ownership, cat	232 (13.7)	128 (14.8)	104 (12.6)	0.191
Pet ownership, dog	393 (23.3)	218 (25.2)	175 (21.2)	0.052
Pet ownership, cat and dog	517 (30.6)	285 (32.9)	232 (28.1)	0.031

There were 37 (2.2%) children with doctor-diagnosed asthma. Among children who were not diagnosed with asthma, a higher proportion of children from biomass fuel households had possible asthma (Table 3; 27.7 vs. 22.2%). The prevalence of asthma and current wheeze did not differ significantly between the 2 groups.

**Table 3:** Respiratory outcomes among children based on household cooking fuel type

	Overall (*n* = 1690)	Biomass fuel households (*n* = 865)	No biomass fuel households (*n* = 825)	*P* value
Respiratory outcomes, *n* (%)				
Diagnosed asthma				
Yes	37 (2.2)	20 (2.3)	17 (2.1)	0.724
No	1653 (97.8)	845 (97.7)	808 (97.9)	
Possible asthma				
Yes	413 (24.4)	234 (27.7)	179 (22.2)	0.009
No^a^	1240 (73.4)	611 (72.3)	629 (76.8)	
Current wheeze				
Yes	156 (9.2)	82 (9.5)	74 (9.0)	0.676
No	1514 (89.6)	771 (89.1)	743 (90.1)	
Missing	20 (1.2)	12 (1.4)	8 (0.9)	

^a^Children with reports of no physician-diagnosed asthma and ascertained to have no possible asthma

Among children with current wheeze (Table 4; *n* = 156), the authors also assessed the prevalence of night and daytime symptoms of asthma and the degree of severity of asthma symptoms. The use of biomass fuels for cooking was not statistically related to number of wheezing episodes, waking at night due to wheezing symptoms, or speech limitations due to symptoms. However, a higher proportion of children from biomass fuel households were classified as having symptoms of severe asthma compared to children from cleaner fuel households (18.2 vs. 7.6%; *p* = 0.048).

**Table 4:** Prevalence and severity of symptoms among children with current wheeze by household fuel status (*n* = 156)

	Biomass fuel households (*n* = 77)	No biomass fuel households (*n* = 79)	*P* value
Wheeze frequency, *n* (%)			
< 4 attacks per week	64 (83.1)	68 (86.1)	0.609
≥ 4 attacks per week	13 (16.9)	11 (13.9)	
Sleep disturbance due to wheeze, *n* (%)			
< 1 night of sleep per week	53 (68.8)	58 (73.4)	0.527
≥ 1 night of sleep per week	24 (31.2)	21 (26.6)	
Difficulty in completing sentence, *n* (%)			
No speech limitation	49 (63.6)	49 (62.0)	0.835
Speech limit to 1–2 words per breath	28 (36.4)	30 (38.0)	
Symptoms severity categories, *n* (%)			
Mild/moderate persistent	63 (81.8)	73 (92.4)	0.048
Severe persistent	14 (18.2)	6 (7.6)	

In univariate analysis, possible asthma and symptoms of severe asthma were positively associated with household biomass fuel types (Table 5). Similar positive associations were observed for mother’s education levels, and pet ownership for possible asthma, but not for symptoms of severe asthma. After adjusting for potential confounders, the association between biomass fuel use and symptoms of severe asthma remained statistically significant, but the association for possible asthma only trends towards significance (*p* = 0.125). In addition, the associations between mother’s education levels, and pet ownership for possible asthma (not symptoms of severe asthma) remained significant. The association between sex and possible asthma was also significant but this was confined to male children only (male: OR = 1.51; 95%CI: 1.04–2.19; female: OR = 1.00; 95%CI: 0.70–6.35). However, the gender-based effects were not seen for symptoms of severe asthma (male: OR = 2.30; 95%CI: 0.67–7.89; female: OR = 2.44; 95%CI: 0.93–6.35). Data not shown in table.

**Table 5:** Associations between personal and environmental characteristics and respiratory health outcomes and severity among the study population (*n* = 1690)

	Possible asthma (*n* = 413)		Symptoms of severe asthma^b^ (*n* = 156)	
	Univariate Model OR (95%CI)	Adjusted Model* OR (95%CI)	Univariate Model OR (95%CI)	Adjusted Model^c^ OR (95%CI)
Household fuel type				
No biomass fuel	1.00	1.00	1.00	1.00
Biomass fuel	1.35 (1.08–1.68)^*a*^	1.22 (0.95–1.56)	1.92 (1.02–3.61)^*a*^	2.37 (1.16–4.84)^*a*^
Sex				
Male vs. female	0.82 (0.65–1.02)	0.83 (0.39–0.88)^*a*^	1.00 (0.53–1.87)	1.14 (0.55–2.37)
Age (per year)	0.96 (0.92–1.00)	0.95 (0.91–1.00)	0.94 (0.83–1.07)	0.95 (0.81–1.10)
Mother education				
< High school	1.00	1.00	1.00	1.00
≥ High school	0.54 (0.36–0.80)^*a*^	0.58 (0.39–0.88)^*a*^	0.70 (0.23–2.15)	0.65 (0.20–2.06)
Pet ownership				
Yes vs. no	1.50 (1.18–1.89)^*a*^	1.57 (1.20–2.04)^*a*^	1.11 (0.57–2.17)	1.03 (0.48–2.18)

^*a*^ *P* < 0.05

^b^Symptoms of mild/moderate persistent asthma was used as reference category

^c^Model adjusted for each variable in the table as well as body mass index and environmental tobacco smoke exposure

## Discussion

The use of solid fuels such as wood, charcoal, dried animal dung and agricultural residues, as sources of domestic cooking energy has, recently, received great attention. The ISAAC Phase 3 study reported that the highest prevalence of symptoms of severe asthma (among wheezing children) was found in the LMICs [26]. Factors responsible for this high prevalence are still not well evaluated.

In the current study, we demonstrated that household biomass fuel use may worsen asthma conditions in rural children with asthma or asthma-like symptoms. Wheezing children from households using biomass fuels for cooking experienced more severe asthma symptoms compared to children from households using cleaner fuels. Findings from this study also reveal that despite a lower prevalence of diagnosed asthma among children from both biomass fuel groups, a higher proportion of children were classified as having possible asthma in both groups (which is significantly higher in children from biomass fuel group). This suggests that majority of children in the studied rural communities may probably have asthma, but are either unaware of their asthma conditions or have not yet been diagnosed by a healthcare professional as having asthma.

Several features of the rural environment (including socio-economic status [SES], access to healthcare, dust exposure) have been observed to worsen asthma condition and symptoms severity in children [24, 27]. This study provides further evidence to support a connection between biomass fuel use and the exacerbation of asthma symptoms in rural settings where the practice is predominant.

Few studies to date have investigated the effect of HAP on asthma symptoms severity as the main focus, having instead chosen to investigate the prevalence and incidence of asthma. In a cross-sectional study among 1,058 children in Guatemala, the use of open fire for cooking was associated with an increased risk of wheezing episode, night sleep disturbance due to wheeze, and speech limitation between breaths due to wheezing symptoms, albeit non-significantly except for speech limitation [28]. The findings from the current study confirmed those of the Phase III global investigation of childhood asthma prevalence by the ISAAC team, where the use of open fire was observed to increase the risk of current symptoms of severe asthma in children 6–7 years old (aOR = 1.79; 95%CI: 1.18–2.70) [9]. However, in the ISAAC study, the report of open fire for cooking was 0 and 18% for children aged 6–7 years and 13–14 years, respectively, in the African region; as such, its findings could not be generalized to the African region. The study at hand demonstrates a possible link between the use of open fire and symptoms of severe asthma, but in a different rural African population that primarily cook with biomass fuel. Although studies have shown that rural environment may confer some protections against the development of asthma in children [29–32], results of this study further demonstrate that some rural areas may have certain environmental factors that place children at the same risk for development of asthma; and once children are afflicted with asthma or have asthma symptoms, certain rural exposure may aggravate the severity of their respiratory conditions. Therefore, regardless of the actual prevalence of asthma in the population, our study further supports the concept that children from rural settings or LMICs with exposure to biomass fuel may be at increased risk for more severe asthma.

Overall, a 24% prevalence of possible asthma (unassociated with a diagnosis) was observed, suggesting that the true asthma prevalence in this population may be higher than previously known due to possible underdiagnosis of asthma among rural children. Although urban children were not recruited in this study, the findings show a similar trend to observations from a study in the USA where children from rural settings were less likely to be diagnosed with asthma and more likely to be characterized as “at-risk” of asthma (27.8%) compared to urban children (24.6%) [33].

Studies have revealed some factors that may contribute to asthma underdiagnosis among children, especially rural children, including poor access to healthcare for symptoms reporting and diagnosis [34] and/or diagnostic differences in asthma recognition and labelling by physicians [35]. In the present study, the authors do not feel that the reason for underdiagnosis among children in these rural settings was due to diagnostic differences in asthma recognition because the survey instrument used was translated from English to the Yoruba language and reverse-translated by expert translators for clarity and better understanding of symptoms; it was also piloted by the authors in similar rural communities [16]. Instead, it may be due to lack of symptoms awareness among parents and accessibility to healthcare centers, which could signify target interventions for children’s respiratory health in these communities. In the ISAAC studies, the overall prevalence of asthma symptom (current wheeze) among children in Nigeria has been observed to have increased between Phase 1 and Phase 3 of the study (4.5 to 5.6%, respectively, for 6–7 year age-group and 10.7 to 13%, respectively, for the 13–14 year age-group) [36]. One of the explanations for the apparent increase was a greater awareness of asthma-related symptoms, especially in the LMICs. However, the relatively low asthma prevalence observed in our study population, despite a higher prevalence of asthma-related symptoms, suggests improved efforts to increase awareness about asthma symptoms, especially wheezing (which may be a subjective complaint that could be described and interpreted in several ways), among rural populations in Nigeria. This could improve symptoms reporting, better diagnosis of asthma and provide accurate estimate of the disease prevalence among children in Nigeria.

We must consider limitations of our study. First, since households can use a combination of fuel types, there could be possibility of exposure misclassification which may inflate our results. However, in the survey questionnaire, parents had the options to choose from different energy sources: “cow dung/animal residue,” “firewood,” “charcoal,” “liquefied petroleum gas (LPG),” “electricity,” and “other. If parents chose “other,” they also need to specify which fuel they used. To minimize exposure misclassification, only children from parents using cow dung/crop residue, firewood, charcoal, or a combination of two or more of these household fuel types were classified as children from households using biomass fuels. Moreover, the survey questionnaire was translated from English to Yoruba (the commonly spoken language in the community) for easy understanding by the parents. Therefore, while it may be difficult to completely eliminate exposure misclassification in this study, we would expect that this would occur non-differentially between the biomass fuel user groups allowing our interpretation of the results comparing the two groups to remain valid. Another limitation of this study is the lack of information on household SES and accessibility to healthcare, which could be related to asthma symptoms severity. It is possible that many of the children with severe symptoms of asthma were neither involved in cooking activities nor has at any point in time being exposed to biomass smoke. Symptoms severity might have occurred due to lack of access to healthcare centers for symptoms diagnosis or lack of appropriate symptoms management due to parental financial constraints. Such information would have strengthened the results of the study by enabling the separation of current asthma symptoms that are related to chronic exposure to biomass fuel from that associated with family SES. However, because mothers’ education level, used as proxy indicator of SES in the multivariate analysis, was not significant in this study, we do not believe other SES indicators would have changed our results significantly since the rural populations involved in this study are mostly homogeneous. Finally, we did not recruit children from urban centres in this study. Although, we believe that some households in urban centers in Nigeria also use biomass fuels in combination with other cleaner fuels (e.g., LPG, kerosene and electricity) for cooking and energy needs, we limited recruitment of our participants to communities that are predominantly homogeneous in terms of socioeconomic status, home construction, and access to health care to minimize residual confounders in our results. However, whether the patterns of risk of symptoms of severe asthma observed in rural children exposed to biomass fuels in the current study mirror similar pattern in urban children, with varied household characteristics, or the risk could be mediated by a combination of other factors warrants further investigation.

Large sample size and the use of standardized and validated instruments across all rural regions included in this study are the strengths of this study. This allowed us adequate statistical power to consider associations between asthma severity and biomass fuel, as part of the rural population exposure characteristics. Also, the standardized methods of assessment were identical in all locations and biomass fuel user groups so that the observed differences could not be attributed to methodological discrepancies.

## Conclusion

The current study demonstrated worse asthma symptoms among children exposed to HAP from burning solid fuels and possible asthma underdiagnosis in rural Nigerian schoolchildren. Children from these rural settings were also more likely to be have possible asthma and less likely to be diagnosed as asthmatic. The study further highlights the role of biomass fuel use as a potential risk factor for increasing asthma morbidity in rural Nigerian settings and provides an opportunity for utilizing schools to identify children at risk for asthma. It is currently impossible to eliminate the use of solid fuels as sources of domestic cooking energy in rural communities in Nigeria. Therefore, findings from this study call for integrated interventions to reduce HAP in order to improve lung health among children and a need for school-based health centers for childhood asthma screening and diagnosis. However, until such an upstream intervention can be achieved, avoidance of factors that could trigger and/or worsen asthma, such as the reduction of exposure to biomass fuel smoke from cooking, may form a crucial component of asthma management guidelines for children in rural Nigeria.

## Abbreviations

BMI: Body Mass Index
CI: 95% confidence interval
HAP: Household Air Pollution
ISAAC: International Study of Asthma and Allergies in Childhood
LMICs: Low- and Middle-Income Countries
LPG: Liquefied Petroleum Gas
OR: Odds ratio
